# Influenza Activity — United States, September 30–December 1, 2018

**DOI:** 10.15585/mmwr.mm6749a4

**Published:** 2018-12-14

**Authors:** Alicia P. Budd, Anwar Isa Abd Elal, Noreen Alabi, John Barnes, Lenee Blanton, Lynnette Brammer, Erin Burns, Charisse N. Cummings, Vivien G. Dugan, Shikha Garg, Rebecca Garten, Lisa A. Grohskopf, Larisa Gubareva, Krista Kniss, Natalie Kramer, Alissa O’Halloran, Wendy Sessions, Calli Taylor, David E. Wentworth, Xiyan Xu, Alicia M. Fry, Jacqueline Katz, Daniel Jernigan

**Affiliations:** 1 Influenza Division, National Center for Immunization and Respiratory Diseases, CDC.

## Abstract

Influenza activity in the United States was low during October 2018, and, although it increased slowly during November, activity remains low across most of the country.* During the week ending December 1, 2018, the percentage of outpatient visits for influenza-like illness^†^ (ILI) was equal to the national baseline^§^ (Figure) and was at or slightly above the region-specific baseline in four of the 10 U.S. Department of Health and Human Services regions^¶^ (Regions 4 and 7–9). The majority of jurisdictions experienced minimal or low ILI activity since September 30; however, two experienced moderate ILI activity, and two experienced high ILI activity** during the week ending December 1. The percentage of deaths attributed to pneumonia and influenza remains below the epidemic threshold,^††^ and the rate of influenza-associated hospitalizations remains low. Five laboratory-confirmed, influenza-associated pediatric deaths occurring since September 30 have been reported to CDC. During the week ending December 1, the majority of jurisdictions (40 states, the District of Columbia, Puerto Rico, and U.S. Virgin Islands) reported sporadic or local geographic spread of influenza activity, nine states reported regional activity, and one state reported widespread activity.^§§^

**FIGURE:**
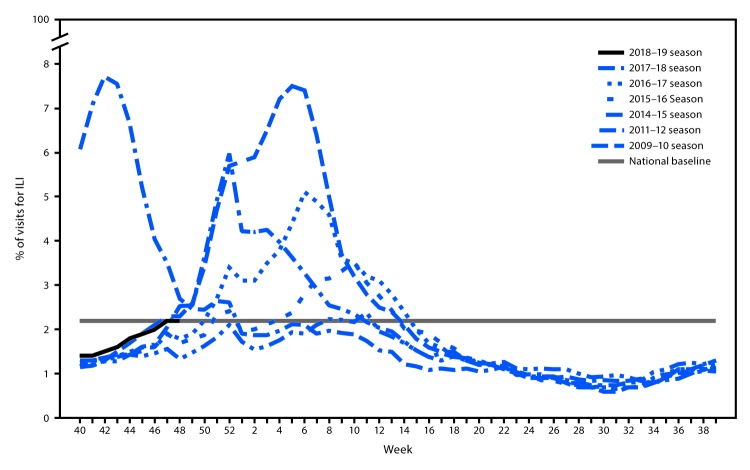
Percentage of visits for influenza-like illness (ILI) — U.S. Outpatient Influenza-like Illness Surveillance Network (ILINet), weekly national summary, 2018–2019* and selected previous seasons * As of December 7, 2018.

Influenza A(H1N1)pdm09 viruses have been reported most frequently (67% of all viruses and 81% of subtyped influenza A viruses) by U.S. public health laboratories since September 30, 2018 (Table), but A(H3N2) and influenza B viruses also were reported. The majority of influenza viruses characterized during this period were genetically and antigenically similar to the cell-grown reference viruses representing the 2018–19 Northern Hemisphere influenza vaccine viruses.^¶¶^ No viruses with resistance to oseltamivir, zanamivir, or peramivir have been identified.

**TABLE:** Influenza positive specimens reported by U.S. public health laboratories — United States, September 30–December 1, 2018*

Influenza virus type/Subtype or lineage	No. of positive specimens (% of total)
**Influenza A viruses**	
Influenza A(H1N1)pdm09	740 (67)
Influenza A(H3N2)	176 (16)
Influenza A (subtyping not performed)	92 (8)
**Influenza B viruses**	
Influenza B Yamagata	60 (5)
Influenza B Victoria	21 (2)
Influenza B (lineage not performed)	22 (2)

* As of December 7, 2018.

The timing of influenza activity often varies; however, influenza activity will increase in coming weeks and is likely to peak during December–February. Annual influenza vaccination is recommended for all persons aged ≥6 months who do not have contraindications (*1*). Multiple influenza vaccines are approved and recommended for use during the 2018–19 season, and vaccination should continue to be offered as long as influenza viruses are circulating and unexpired vaccine is available. For the 2018–19 season, manufacturers projected they would supply the United States with 163–168 million doses of influenza vaccine. As of November 30, 2018, approximately 163.8 million doses had been distributed.

Influenza antiviral medications are an important adjunct to vaccination in the treatment and prevention of influenza. There are four recommended influenza antiviral medications for treatment of influenza this season: oral oseltamivir, inhaled zanamivir, intravenous peramivir, and the newly approved oral baloxavir. Treatment with influenza antiviral medications as close to the onset of illness as possible is recommended for patients with confirmed or suspected influenza who have severe, complicated, or progressive illness; who require hospitalization; or who are at high risk for influenza complications. Some antiviral medications (oseltamivir and zanamivir) can be considered for chemoprophylaxis to prevent influenza in certain situations; however, general seasonal or preexposure antiviral chemoprophylaxis is not recommended. Updated recommendations for use of antiviral drugs are available (https://www.cdc.gov/flu/professionals/antivirals/summary-clinicians.htm).

Influenza surveillance reports for the United States are posted online weekly (https://www.cdc.gov/flu/weekly). Additional information regarding influenza viruses, influenza surveillance, influenza vaccines, and influenza antiviral medications is available online (https://www.cdc.gov/flu).

## Footnotes

* Data as of December 7, 2018.

¶¶ The recommended Northern Hemisphere 2018–19 trivalent influenza vaccine composition includes an A/Michigan/45/2015 (H1N1)pdm09-like virus, an A/Singapore/INFIMH-16-0019/2016 (H3N2)-like virus, and a B/Colorado/06/2017-like (Victoria lineage virus), with an additional influenza B virus (B/Phuket/3073/2013-like [Yamagata lineage]) virus recommended for quadrivalent vaccines.
